# Patient care ownership in medical students: a validation study

**DOI:** 10.1186/s12909-023-04106-6

**Published:** 2023-02-22

**Authors:** Tasha R. Wyatt, Elena A. Wood, Jennifer L. Waller, Sarah C. Egan, Lara M. Stepleman

**Affiliations:** 1 Center for Health Professions Education, Department of Medicine, Uniformed Services University of the Health Sciences, 4301 Jones Bridge Road, Bethesda, MD 20814-4712 USA; 2 Office of Academic Affairs, Medical College of Georgia at Augusta University, Augusta, GA USA; 3 Department of Population Health Science, Division of Biostatistics & Data Science, Medical College of Georgia at Augusta University, Augusta, GA USA; 4 Department of Psychiatry and Health Behavior, Medical College of Georgia at Augusta University, Augusta, GA USA

**Keywords:** Patient care ownership, Instrument, Validity, Undergraduate medical education, Medical students

## Abstract

**Background:**

Psychological Ownership is the cognitive-affective state individuals experience when they come to feel they *own* something. The construct is context-dependent reliant on *what* is being owned and by *whom.* In medical education, this feeling translates to what has been described as “Patient Care Ownership,” which includes the feelings of responsibility that physicians have for patient care. In this study, we adapted an instrument on Psychological Ownership that was originally developed for business employees for a medical student population. The aim of this study was to collect validity evidence for its fit with this population.

**Methods:**

A revised version of the Psychological Ownership survey was created and administered to 182 medical students rotating on their clerkships in 2018–2019, along with two other measures, the Teamwork Assessment Scale (TSA) and Maslach Burnout Inventory (MBI) Survey. A confirmatory factor analysis (CFA) was conducted, which indicated a poor fit between the original and revised version. As a result, an exploratory factor analysis (EFA) was conducted and validity evidence was gathered to assess the new instruments’ fit with medical students.

**Results:**

The results show that the initial subscales proposed by Avey et al. (i.e. *Territoriality, Accountability, Belongingness, Self-efficacy, and Self-identification*) did not account for item responses in the revised instrument when administered to medical students. Instead, four subscales (*Team Inclusion, Accountability, Territoriality*, and *Self-Confidence*) better described patient care ownership for medical students, and the internal reliability of these subscales was found to be good. Using Cronbach’s alpha, the internal consistency among items for each subscale, includes: *Team Inclusion* (0.91), *Accountability* (0.78), *Territoriality* (0.78), and *Self-Confidence* (0.82). The subscales of *Territoriality, Team Inclusion*, and *Self-Confidence* were negatively correlated with the 1-item Burnout measure (P = 0.01). The *Team Inclusion* subscale strongly correlated with the Teamwork Assessment Scale (TSA), while the subscales of *Accountability* correlated weakly, and *Self-Confidence* and *Territoriality* correlated moderately.

**Conclusion:**

Our study provides preliminary validity evidence for an adapted version of Avey et al.’s Psychological Ownership survey, specifically designed to measure patient care ownership in a medical student population. We expect this revised instrument to be a valuable tool to medical educators evaluating and monitoring students as they learn how to engage in patient care ownership.

## Introduction

“Taking ownership” is a commonly used phrase in medical education to communicate physicians’ responsibility and commitment to patients and their care. The phrase is ubiquitously used in clinical settings and often interchanged with variations that include, “decision ownership”, “psychological ownership”, and “patient care ownership” [1]. In all of its uses, ownership references the cognitive-affective state that develops as a result of individuals getting to know something (e.g., a patient), managing it (e.g. making decisions, medication management, etc.), and then emotionally investing in a relationship [2].

Although the phrase remains in common use, some researchers have argued that it is outdated because it originated during a time when physicians spent so much time in the hospital caring for patients that they eventually came to *own* them [3]. And yet, medical educators continue to use it today because it serves as a shortcut to communicate other aspects of clinical care, including *responsibility, commitment, advocacy*, and *personal investment* [4], all of which are critical to high quality patient care.

Students typically begin to hear this phrase in their third and fourth years when attending physicians and clerkship directors set ownership as an expectation during their clerkship rotations [5, 6]. Ownership for these students, in this setting, starts at the periphery of direct patient care while students are learning how to be a physician. For instance, in their third and fourth years, medical students often play the role of observer rather than caretaker, and similarly, they engage in shadowing other physicians, which by definition means that the student is in the background and not directly involved in the provision of care. In other cases, students are relegated to doing “scut work,” in which they take care of paperwork, phone calls, and other duties busy physicians don’t have time to do. All of these roles are important in learning to be a physician, but do not include taking care of patients as the central task.

As such, several studies have investigated the ways in which ownership can be developed in medical students, including how various clinical settings facilitate and constrain students’ engagement in these behaviors [7], and how clerkship directors create learning environments that support students to take ownership [8]. Other researchers have investigated how taking ownership of patients’ care works as a catalyst for triggering professional identity formation experiences in a simulated environment [5, 9].

However, the majority of research has been conducted in residency training where trainees are expected to exhibit these behaviors. For example, researchers have created interventions that help to identify who has responsibility in patient handoffs [10] and how duty-hour restrictions have changed care-taking behaviors [3]. Other studies have attempted to define ownership within various specialties, demonstrating how the myriad definitions intersect and differ depending on the tasks performed [11–13]. And still others have advocated for the inclusion of patient perspectives to create a broader understanding of what is expected of physicians as they take ownership of patients and their care [14].

To date, only one study has attempted to develop an instrument to measure this construct in medical education. Developed by Djulbegovic et al. [15], this instrument designed a measure to assess feelings of psychological ownership in residents in hopes of improving their ability to care for patients. The Djulbegovic et al. instrument used Avey et al.’s [16] construct of psychological ownership as a foundation, and then supplemented it with constructs and themes identified by the team’s earlier studies [12, 17]. While Djulbegovic et al. [15] instrument is appropriate for residents, it is not useful for medical students because it includes items that inquire into tasks that are only available to residents who are further along in their training (i.e., clinical decision-making, medication management, etc.).

Therefore, we created a measure of patient ownership for medical students using Avey’s original measure. Our rationale for doing so was that students are expected to take ownership of patients at the level commensurate with their training [6] and Djulbegovic’s scale [15] was too advanced for medical students. However, we wanted to be consistent with their construct to maintain consistency and continuity through the training environment. Using Downing’s [18] framework, which combines theory, predicted relationships, and empirical evidence, we provide validity evidence for a new instrument measuring patient ownership in medical students to be used during their clerkship rotations.

## Materials and methods

Downing’s [18] model is an extension of Messick’s validity framework [19], which argues that assessments are not valid in and of themselves; rather they are the result of researchers’ evidence gathered in support of a specific interpretation. As such, we position this study as an argument for the validity evidence collected on the adaptation of Avey et al.’s [16] measure of *psychological ownership*, a construct that originates in organizational psychology to describe the feeling of responsibility individuals have for the tasks, processes, or people within their sphere of influence and control. We chose to adapt this instrument because, like employees who feel that various projects belong to them, physicians share similar feelings about their patients and their care [20]. Further, we understand that while medical students are not expected to take ownership of their patients’ care, they are expected to develop these feelings while in training and are granted access to patients in a limited manner to learn how to do so.

According to Downing [18], researchers arguing for validity must provide scientifically sound evidence within five components: content, response process, internal structure, relationship to other variables, and consequences. In using these components, he frames the validation process as an argument in which each new study adds validity evidence and demonstrates further evidence for the instrument’s strengths and weaknesses within the types of validity evidence it is best designed to capture. We used Downing’s model to frame our work because it is widely accepted as a model for collecting validity evidence in medical education [21–24].

### Phase I: scale development

We were granted permission to use Avey et al.’s [16] original instrument by MindGarden in 2014, which contained 16 items and five subscales. When these five subscales are combined, they are thought to coalesce in a measure of ownership. The subscales are: (a) *Territoriality* - when individuals feel they must mark their place or objects, believing they have exclusive rights to them; (b) *Accountability -* the expectation that one may be asked to justify one’s beliefs, feelings and actions; (c) *Self-Efficacy –* the idea that people’s beliefs facilitate or constrain success as they attempt to implement action or complete a specific task; (d) *Belongingness* - the psychological need individuals have for feeling they have a home or place; and (e) *Self-Identification* - when individuals internalize the organizational identity as an extension of one’s self. Previous studies had noted how these various subscales rank in clerkships as students develop ownership over their patients and their care [8].

Avey et al.’s [16] original instrument prompted respondents to comment on 16 items using a 6-point Likert scale ranging from “Strongly Disagree [1] to Strongly Agree [6].” The validity evidence for the original version of the instrument included moderate to good coefficient alphas for the all subscales: Self-Efficacy (α = 0.90), Accountability (α = 0.81), Belongingness (α = 0.92), Self-Identification (α = 0.73), and Territoriality (α = 0.84), and good relationship to other meaningful constructs such as Transformational Leadership, Organizational Citizenship Behavior, Organizational Commitment, Workplace Deviance, Intentions to Stay, and Job Satisfaction [16].

Our team, which has expertise in the conceptual and theoretical underpinnings of patient care ownership, medical education, and survey validation, modified the items to fit within a medical education setting. After adapting the items from business to medical education, the newly modified instrument was piloted in 2016 at the Medical College of Georgia with third- and fourth-year students to assess feasibility and relevancy for a medical school population [7]. Further language modifications were made to be more inclusive of healthcare teams. For example, if the original item was worded as “I feel I belong in this organization,” it was originally written, the item was changed so that it read “I feel I belong on this healthcare team.”

Each revised item was then considered for various interpretations (to ensure clarity) and whether these interpretations would ensure the same construct is being asked about in the original item. To establish content validity, it was then sent to clerkship directors for feedback. Once received, the team reviewed the original instruments used to create Avey et al.’s [16] survey and added additional items to four of the five subscales to include a more complete understanding of psychological ownership in healthcare settings. For example, for the *Accountability* subscale in the original Avey et al. study, we added the following item: “I consistently hold myself accountable for my patients’ care.” Additionally, for the *Self-Identification* subscale, we added “I have difficulty using my knowledge in patient care on this healthcare team” (reversed item).

The items in the *Territoriality* subscale remained at 4 items, while items in the other scales all increased from the original 3 items: *Accountability* (5 items), *Self-Efficacy* (6 items), *Belongingness* (5 items), and *Self-Identification* (7 items). All changes were incorporated into the instrument and the survey was renamed the *Patient Ownership Survey.*

To collect validity evidence on the *Patient Ownership Survey’s* relationship to other variables, we also included two other measures in our validation process; the Teamwork Assessment Scale (TSA) [25] and the 1-item Maslach Burnout Inventory (MBI)-Human Services Survey [26]. The TSA was previously validated in a medical student population [25] and measures individuals’ level of teamwork within a specific setting. We included this survey because in our pilot study, we found that a sense of belongingness, which is one of Avey et al.’s [16] original subscales had a strong correlation with students’ willingness to take ownership of their patients [7]. The TSA is comprised of three sub-scales measuring team adjustment behaviors, team coordination and cooperation, and information exchange.

The other measure included with our *Patient Ownership Survey* was the Maslach Burnout Inventory (MBI)-Human Services Survey, which is a one-item shortened version of the full Burnout scale [26]. The scores on this instrument range from 1 to 5, with higher scores indicating a lower level of burnout. We included these other two measures because we hypothesized that teamwork would be positively associated with aspects of psychological ownership and negatively associated with burnout, as others have suggested [15, 27].

### Phase II: psychometric evaluation of the modified patient care ownership

The adapted *Patient Ownership Survey* included 27 items, which was an 11 item increase from the 16 items included in Avey et al.’s [16] original instrument; it was not anticipated that all new items might function equally well within the scale and some might be dropped. The survey also included questions to collect demographic information (i.e., age, gender, ethnicity/race) and descriptions of the medical education setting of respondents (i.e., clerkship, year in medical school, campus, clinical setting). Demographic information was used to collect validity evidence on relationships with other variables. Based on our previous research, variables like type of clerkship and clinical settings can influence the level of patient care ownership [7, 8]. This data was also used to collect validity evidence.

### Study design and setting

The adapted instrument was distributed to third-and fourth-year students through the Medical College of Georgia’s secure online evaluation system. Participants included third- and fourth-year medical students enrolled in the 2018–2019 school year who were rotating through their clerkships. Third year clerkships included: Internal Medicine, Family Medicine, Obstetrics and Gynecology (OB/GYN), Surgery, Pediatrics, Neurology, and Psychiatry. Fourth-year rotations included Emergency Medicine, Ambulatory Medicine, and various electives.

To collect consequential validity evidence on how patient ownership may develop over time, and to recruit as many students as possible into the study with at least two data points, we sent the survey out several times. In Downing’s framework [18], consequential validity is concerned with whether an experience, in this case clerkships, creates change over time, and is different from test-retest reliability, which assesses whether the construct is stable over time. Therefore, during the academic year 2018-19 the survey was sent approximately three times three months apart to third year and only once in the spring to fourth year students.

Participation in this study was voluntary and all students provided full and informed consent prior to participating. To document response process, trained researchers kept a decision journal on the challenges and questions they encountered in the writing of items, administration of the survey, and interpretation of the results. These entries were used to continually adjust the items throughout the validation process, and track where we experienced challenges in the development of the new instrument. The study was approved by the Medical College of Georgia’s University’s Institutional Review Board (Protocol #920,339).

### Statistical analysis

All statistical analysis were performed using SAS 9.4 and statistical significance was assessed using an alpha level of 0.05. Descriptive statistics on all variables were determined overall, and by measurement time where appropriate.

In using Downing’s [18] multi-component approach to assessing validity, we prioritized internal structure validity in the analysis. To this end, the team assessed the instrument’s internal structure using confirmatory factor analysis (CFA) by making use of each student’s first administration of the survey. Not unexpectedly given the items instrument revisions, the CFA indicated a poor fit of the revised version of the measure with the original. As a result, an EFA was performed using a principal components extraction method with varimax rotation and Kaiser normalization. The number of factors was determined using eigenvalues > 1, a scree plot, and parallel analysis. Items that loaded on two or more factors or did not load within a minimum factor loading of 0.40 were excluded. Cronbach’s alpha was determined for the overall and sub-scales in the revised ownership scale. Pearson correlations of the overall and sub-scales revised factors were calculated.

To examine relationships between the revised sub-scales and various variables, two-sample t-tests, Pearson correlations, and one-way ANOVA between different racial/ethnic groups with a Tukey-Kramer multiple comparison procedure were examined. Other demographics included age, gender, year in medical school, clinical settings, including identification of the campus, type of clinical setting, and number of students on rotation. Assessing the number of students on the team is important because it has potential to affect feelings of patient care ownership and ownership behavior [7]. Given the literature on patient care ownership and how it develops, the research team expected to find correlations between the adapted Patient Care Ownership Survey and Teamwork Assessment Scale [25], and the Burnout Inventory Survey [26].

To assess consequential validity, mixed models were used to examine whether changes over time were seen for both the original and exploratory sub-scales. The subject was considered the random effect and survey administration time (1, 2, or 3) was considered a fixed effect. Mixed model analysis allows for all available data for a subject to be utilized in an analysis to estimate the adjusted least squares means. All subjects with data at baseline were included in the analysis, even if they had missing data at one or more subsequent follow-up time points. Missing data were not imputed. All data collection and analysis methods were performed in accordance with the relevant guidelines and regulations.

## Results

### Participants

Of the total number of students invited to participate in the study, 182/487 responded, indicating a 37% response rate. Of these 182 students, 118 were in their third year and 64 students in their fourth year. Class cohorts were equally represented in the data set, and students had a mean age of 26.2 years (SD = 2.1). Students indicated that the mean number of students rotating with them on their clerkships was 4.4 students (SD = 6.8). Demographic data also showed that students rotated through various clerkships and clinical settings across the state, thus providing a broad representation of different clinical sites available to students. See Table 1 for extended demographic information.

**Table 1:** Demographic information for medical students participated in the study

Variable	Level	N = 182
Sex – n (%)	Male	83 (45.6)
	Female	99 (54.4)
Race – n (%)	White	94 (51.7)
	Black/African American/African	13 (7.1)
	Asian/Pacific Islander	46 (26.3)
	Hispanic	12 (6.6)
	Multiracial/Unknown	17 (9.3)
Student Year – n (%)	Third year	118 (64.8)
	Forth year	64 (35.6)
Setting – n (%)	Medical Center	92 (51.1)
	Rural Hospital	37 (20.6)
	Community Provider	51 (28.3)

### Internal structure

#### Factor structure

To examine the internal structure, we took a 2-step approach to examining the factor structure of the *Patient Care Ownership Survey* items. The CFA attempted to replicate the initial 5-factor structure; however, this model resulted in a poor fit with our data and fell short on all parameters. The CFA resulted in a statistically significant chi-square test (χ^2^ = 1140.93, p < 0.0001), a RMSEA = 0.1210 (90%CI 0.1135–0.1285), CFI = 0.7027, NFI = 0.1411, and NNI = 0.4477. Because all fit statistics indicated a lack of fit to the original factor structure set out by Avey et al., an exploratory factor analysis was performed.

In the exploratory factor analysis, examination of the pattern coefficients of the first factor did not support a single generalized response factor, and neither did the Scree plot. Factor solutions for 6, 5, 4, and 3 factors were examined, considering the parsimony and theoretical relevance of each solution to the ownership construct. A four-solution fit the data best with 19 of the 27 original items being maintained and accounting for 64% of the variance. Parallel analyses also supported a four-factor solution. The rotated eigenvalues were 4.60, 2.67, 2.48, and 2.26, respectively (Table 2).

**Table 2:** Patient care ownership subscales description (mean and standard deviation at the baseline measure)

Subscales*	Number of Items	Score range	Sample mean	Sample SD	Rotated eigenvalues	Cronbach’s alpha
Team Inclusion	7	7–42	32.9	5.6	4.60	0.91
Accountability	5	5–30	20.7	3.3	2.67	0.78
Territoriality	4	4–24	17.2	3.7	2.48	0.78
Self-Confidence	3	3–18	11.9	3.3	2.26	0.82

* Each item range 1–6 where 1 is “Strongly Disagree” and 6 is “Strongly Agree”

Factor 1 had seven items and was labeled the *Team Inclusion* subscale. It is comprised of three items from *Belongingness* and four from *Self-Identification*; an exemplar item includes “I have close bonds with my healthcare team.” Factor 2 included four items from *Accountability* and one from *Self-Efficacy* and we named this factor *Accountability*. An exemplar item on *Accountability* includes, “I hold myself accountable for my patients’ care.” Factor 3 included all four items from the original Territoriality subscale and thus we kept the *Territoriality* scale name; an exemplar item includes, “I feel I have to assert my role on this healthcare team.” Factor 4 included two items from Self-Identification and one from *Self-Efficacy* and labeled as the *Self-Confidence* scale; an exemplar item includes, “I do not feel sufficiently confident to try different ways of providing patient care.” Table 3 presents the final factor solution.

**Table 3:** Exploratory factor analysis for Patient Care Ownership scale

Items with the original subscale	Factors			
	1	2	3	4
B- I have a sense of belonging in this healthcare team.	0.878	0.125	− 0.054	− 0.088
B- I have close bonds with my healthcare team.	0.849	0.127	− 0.025	− 0.048
SI- I feel like I am a member of the healthcare team.	0.828	0.122	− 0.047	− 0.210
SI- I am an active member of the healthcare team.	0.788	0.144	0.010	− 0.200
SI- Being a member of this healthcare team is important to me.	0.732	0.199	− 0.051	− 0.057
B- I feel included on this team.	0.728	0.163	− 0.119	− 0.109
SI- I enjoy working as a team.	0.640	0.203	− 0.210	0.085
A- I have the right to hold others accountable for the care of my patient.	0.107	0.786	0.179	− 0.012
A- I have the right to ask for others to justify their management decisions when it pertains to my patients.	0.085	0.778	0.164	0.098
A- I consistently hold myself accountable for my patients’ care.	0.213	0.677	− 0.044	− 0.181
A- I have a right to know what is going on with my patient.	0.226	0.639	− 0.082	0.042
SE- When I make plans for patient care, I am certain I can make them work.	0.306	0.568	0.201	− 0.158
T- I feel I need to protect my work (i.e. notes, slides, records) from others on my healthcare team.*	0.035	− 0.027	0.837	0.216
T- I feel I need to protect my ideas about patients from my peers.*	− 0.075	− 0.002	0.827	0.243
T- I feel that people I work with should not invade my areas of responsibility.*	− 0.098	0.169	0.748	0.010
T- I feel I have to assert my role on this healthcare team.*	− 0.156	0.141	0.574	0.075
SE- I feel insecure about my ability to do things related to patient-care.*	− 0.037	− 0.090	0.077	0.842
SI- I do not feel sufficiently confident to try different ways of providing patient care.*	− 0.241	− 0.110	0.183	0.821
SI- I have difficulty using my knowledge in patient care on this healthcare team.*	− 0.157	0.106	0.307	0.760

Extraction Method: Principal Component Analysis

Rotation Method: Varimax with Kaiser Normalization

a. Rotation converged in 6 iterations

*Reversed item

Modified Avey’s subscales: B – Belongingness, A – Accountability, T – Territoriality, SI – Self-Identification, SE – Self-Efficacy

#### Internal consistency

Using Cronbach’s alpha, the internal consistency among items for each of the *Patient Care Ownership Survey* subscales includes: *Team Inclusion* (0.91), *Accountability* (0.78), *Territoriality* (0.78), and Self-Confidence (0.82). Cronbach’s alphas were acceptable to very good for all subscales items (all alphas≥0.77). For three of the four subscales there was no improvement in internal consistency with removal of any items. For *Territoriality*, removing one of the four items would have improved the internal consistency to 0.80, however, given the exploratory nature and the relatively minor improvement, we chose to keep the four-item scale intact.

Subscale correlations were found with *Territoriality* being positively correlated with *Team Inclusion* and *Self-Confidence*, and negatively correlated with *Accountability*. *Accountability* was positively correlated with *Team Inclusion*, and there was no statistically significant correlation with *Self-Confidence*. *Team Inclusion* was positively correlated to *Self-Confidence* (Table 4). Combined, these correlations provide evidence against summing all the scores across subscales for a single Patient Care Ownership score, which is not uncommon when you are measuring different aspects of a constructInstead, the findings suggest using the sub-scales independently to capture the different aspects of ownership is a more appropriate approach.

**Table 4:** Correlations between Patient care ownership sub-scales and with TSA and Burnout

	Accountability	Team Inclusion	Self-Confidence	TSA	Burnout
Territoriality	-0.30 (< 0.01)	0.15 (0.04)	0.35 (< 0.01)	0.20 (0.01)	-0.11 (0.13)
Accountability		0.18 (0.02)	-0.06 (0.45)	0.05 (0.49)	0.01 (0.85)
Team Inclusion			0.28 (0.0002)	0.55 (< 0.01)	-0.27 (0.01)
Self-Confidence				0.32 (< 0.01)	-0.24 (0.01)
TSA					-0.19 (0.01)

#### Relationship to other variables/construct validity

To gather validity evidence for the relationship of the *Patient Care Ownership Survey* to other variables, we ran Pearson correlations (Table 4) between the instrument’s subscales, and *Teamwork Assessment* (TSA) and Burnout. The *Territoriality, Team Inclusion* (P = 0.01), and Self-Confidence (P = 0.01) subscales were negatively correlated with Burnout. The *Team Inclusion* subscale strongly correlated with TSA, while the subscales of *Accountability* correlated weakly, and *Self-Confidence* and *Territoriality* correlated moderately.

Significant differences on the *Patient Care Ownership Survey* subscales were identified by demographics. In the Accountability subscales, males scored higher than females (Mean ± SD 21.4 ± 3 versus 20.2 ± 3.5, P = 0.02). Overall scores, group included identified as white had higher mean than group who identified as non-white (Mean ± SD = 84.1 ± 9.2 versus 80.3 ± 12.4, P = 0.02). Regarding correlations of the ownership subscales with age, all correlations were low, and while two correlations were statistically significant, and the statistical significance was due to the sample size.

The result of Pearson correlation between Patient Care Ownership subscales with number of medical students on the clerkship indicated statistically significant, but weak correlations with the *Team Inclusion* subscale (P < 0.001).

As described above in the Methods section, we expected the means to differ based on some demographic and clinical settings variables [7, 8].Differences between campuses (regional medical campuses and main campus) significant for *Team Inclusion* ranging between 32.0 and 37.1 on [7–42] range (P = 0.02) and *Self-Confidence* ranging between 11.1 and 14.0 on [3–18] range (P = 0.01) using the Tukey-Kramer multiple comparison procedure. The other two subscales were not statistically significant between the different campuses.

Differences between clinical settings was significant for *Team Inclusion* and *Self-Confidence.* Students rotating at the academic medical center (Mean ± SD = 31.6 ± 5.8) had significantly (P < 0.01) lower scores than those rotating with community providers (Mean ± SD = 34.1 ± 5.4) and rural hospitals (Mean ± SD = 34.1 ± 4.6). For *Self-Confidence*, students at the academic medical center (Mean ± SD = 11.2 ± 3.2) had significantly (p < 0.01) lower scores than those rotating with community providers (Mean ± SD = 13.0 ± 3.1) and rural hospitals (Mean ± SD = 11.8 ± 3.4).

Statistically significant differences (P < 0.01) between clerkships were found in the *Self-Confidence* subscale ranged from 14.3 ± 2.8 (Mean ± SD) in Ambulatory Medicine to 9.9 ± 2.9 (Mean ± SD) in the Surgery clerkship. The Neurology clerkship also had a significantly lower mean score (10.3 ± 3.3) compared to those in Psychiatry (13.9 ± 2.9) clerkship.

#### Consequential

The results of the repeated measures mixed model to examine consequential validity showed that there were no statistically significant differences between measurement times for the ownership subscales or overall scales, except that *Team Inclusion* showed differences between measurement times with Time 1 (Mean (SE) = 32.8 ± 0.4) being significantly (Tukey-Kramer adjusted P < 0.05) lower than Time 3 (Mean (SE) = 34.6 ± 0.6). All subscales and overall scales values increased over time but were not statistically significant.

To examine consequential validity in a cross-sectional way, we compared the results between third- and fourth-year medical students. The fourth-year students (Mean (SD) = 13.0 ± 3.3) had significantly (P < 0.01) higher Self-Confidence score compared to third-year students (Mean (SD) = 11.3 ± 3.2).

## Discussion

Avey et al. [16] argues that the construct of *psychological ownership* is context-dependent and will be expressed differently depending on *what* is being owned, *who* is doing the owning, and other contextual factors shaping the ownership relationship. Using this framing, this study adapted Avey et al.’s [16] instrument for use in medical students where psychological ownership is embedded in the work of medical students at a level commensurate with their ability to contribute to patient care [6].

Our results show that the initial subscales proposed by Avey et al. [16] (i.e. *Territoriality, Accountability, Belongingness, Self-efficacy, and Self-identification*), did not best account for item responses for the revised *Patient Care Ownership Survey.* Instead, four subscales (Team Inclusion, Accountability, Territoriality, and Self-Confidence) better described ownership for medical students, and the internal reliability of these subscales was found to be good. We believe that these adjustments in scale represent the unique experiences of medical students, who are new to the clinical environment, and concerned about both their role and performance on the team. For example, Team Inclusion was important because medical students are new to the medical community, and therefore need to feel included in medicine’s already established teams. As newcomers, students need to feel connected to their team members in ways that go beyond just belonging in the clinical environment [28]. Therefore, our understanding of patient ownership for medical students shifts slightly from earlier conceptualizations on studies with residents [15].

Overall, the current study provided support for the use of this revised patient care ownership instrument, which demonstrated internal structure with a four-factor solution and interpretable factor loadings. Further, the internal consistency of the 19-items of the new instrument sub-scales were good and removing items with low-total correlations did not improve correlations captured in the Cronbach’s alphas. Additionally, we were able to collect support for this new construct presented in the *Patient Care Ownership Survey* through evidence of correlations between subscales, the overall scale, TSA, and Burnout.

Analysis of demographic data shows that our findings are similar to Djulbegovic et al.’s [15] study which did not find that age, gender, inpatient service, patient turnover, supervisory experience, and race to be statistically significant factor in overall patient care ownership scores. However, we believe that these group differences are not theoretically meaningful from a professional development perspective.

We suggest that medical educators use this instrument to track medical students’ skills in developing patient ownership within their clerkships. Specifically, we believe that the new instrument would be useful in longitudinal integrated clerkships where preceptors and students develop relationships over time while caring for patients. Previous research shows that successful precepting practices includes supporting students in taking ownership of patients [29]. And yet, precepting in these clerkships can have a significant impact on preceptors’ time, effort and clinical responsibilities [30]. To alleviate some of the burden, and ensure that students are developing the skills and attitudes needed to take ownership throughout their clerkship experiences, the newly created instrument could be used to supplement the monitoring of students’ development in ways that would support both students and preceptors.

There are several limitations of our study that we encourage other researchers to address as medical education continues to collect validity evidence for the use of the *Patient Care Ownership Survey*. First, the data were collected from students at a single institution, which may indicate sampling bias that would be revealed if tested in another medical school. The 37% response rate was acceptable, but the inclusion of more students in this validation effort would strengthen our argument for the instrument’s use in medical students. Additionally, we cannot know if the students who completed the surveys were different from those who did not; a higher response rate would provide greater response process validity.

The factors need to be re-validated on another medical student population to make sure they hold. Also, future research should examine the new instrument’s relationship to Djulbegovic et al’s [15], which measures psychological ownership in residency, as well as including other variables in the data collection to strengthen validity evidence. This would help substantiate how patient care ownership in medical education is similar and/or different than variables presented elsewhere.

Although there were several limitations in this study, the validity evidence collected here is the first of its kind for measuring patient ownership in a medical student population where early development of this skill is expected. We hope that other researchers engage with the instrument and begin to support students in developing in this important area.

## Data Availability

Our original consent process did not ask permission to post the data set online, Therefore, the datasets used and/or analyzed during the current study are available from the corresponding author upon reasonable request.
